# The safety and immunogenicity to inactivated COVID-19 vaccine in patients with hyperlipemia

**DOI:** 10.1515/med-2023-0780

**Published:** 2023-08-30

**Authors:** Lei Yang, YaMing Liu, Qiao Guo, DePeng Jiang

**Affiliations:** Department of Respiratory Medicine, The Second Affiliated Hospital, Chongqing Medical University, Chongqing, China; Department of Emergency, The Second Affiliated Hospital, Chongqing Medical University, Chongqing, China; Department of General Practice, The Second Affiliated Hospital, Chongqing Medical University, Chongqing, China

**Keywords:** COVID-19 vaccine, safety, effectiveness, hyperlipidemia

## Abstract

It is of urgent need to understand the safety and effectiveness of novel coronavirus (COVID-19)-inactivated vaccine in patients with hyperlipidemia (HLD). However, data on the safety and immune response of SARS-CoV-2-inactivated vaccine in HLD patients are limited. In this prospective study, 105 patients with HLD and 74 healthy controls (HCs) were selected. Within 16–168 days after inoculation-inactivated vaccine, the anti-receptor-binding domain (RBD) IgG and SARS-CoV-2 neutralizing antibodies (NAbs) were evaluated, respectively. Flow cytometry was performed to evaluate RBD-specific B cells and memory B cells. There was no significant difference between HLD patients and HCs in adverse events (AEs) within 7 days after vaccination, and no serious AEs occurred. The seropositivity rates and titers of two Abs (anti-RBD IgG and CoV-2 NAbs) were lower in HLD patients than in HCs (all, *p* < 0.05). HLD showed significantly lower frequencies of RBD-specific B cells than HCs (*p* = 0.040). However, in high cholesterol, high triglyceride, mixed (MiX), and lipid control (HC) subgroups, there was no significant difference in the seropositivity rates and titers of the both Abs. Through mixed factor analysis shows that days between the second dose and sample collection/antibody measurement were associated with the lower anti-RBD IgG antibody levels. In conclusion, inactivated COVID-19 vaccine is safe and well tolerated for HLD patients, but the humoral immune may be limited.

## 1 Introduction

According to the World Health Organization, new coronavirus has wreaked havoc since it first appeared in 2019, causing more than 600 million infections and more than 6 million deaths worldwide as of October 2022. The current novel coronavirus (COVID-19) vaccination remains an effective measure to prevent infection and severe disease, which heavily depends on the production of Abs to SARS-CoV-2 and a sustained immune response. Previous studies have shown that patients with hyperlipidemia (HLD) may increase the incidence of COVID-19 infection and mortality from severe disease [1,2]. The greatest ever study on cholesterol levels was published in the journal *Nature* in 2020, and it revealed that the Chinese population’s cholesterol levels, which may have increased from the lowest in the world, were the highest in the globe, surpassed a number of high-income Western nations [3]. Along with the country’s expanding economy, rising standard of living, and altering lifestyles, the prevalence of HLD is rising quickly in China [4].

According to previous studies, obesity is one of the most common risk factors for thromboembolic events after COVID-19 vaccination [5]; yet, obese patients are often associated with HLD [6]. For the purpose of evaluating the safety of the inactivated COVID-19 vaccine in patients with HLD, we recorded adverse events (AEs) in these patients within 7 days following the immunization.

Although some research have indicated that obesity is not related with an Ab response [7,8], many prior studies [9,10] have demonstrated that obese patients have a low Ab response to the COVID-19 vaccination and the majority of obese patients have impaired lipid metabolism. It is unknown in China whether patients with HLD can produce a potent immune response following an inactivated COVID-19 immunization. Therefore, after administering the COVID-19 vaccine to HLD patients, we evaluated the seropositivity rates and titers of the Abs that were created.

Following vaccination, B-cell immunity plays a major role in the generation of particular Abs. Memory B cells (MBCs), which quickly proliferate and differentiate into antibody-secreting cells when reinfection occurs, mediate persistent humoral immunity [11,12]; this prevents the onset of serious illness or death. The importance of B-cell levels in HLD after vaccination with the COVID-19 vaccine, however, has not yet been studied in detail.

To evaluate the safety and Abs’ response of COVID-19-inactivated vaccine, 105 patients with HLD and 74 healthy controls (HCs) were enrolled in the study. MBCs and receptor-binding domain (RBD)-specific B cells were found using flow cytometry.

## 2 Materials and methods

### 2.1 Population and study design

From May 2021 to December 2021, all participants were enrolled in the study at the Second Hospital Associated with Chongqing Medical University. Inclusion standards was as follows: (1) hypercholesterolemia, which is defined as an elevated serum total cholesterol level greater than 572 mmol/L and a normal triglyceride level, or triglycerides less than 170 mmol/L; (2) hypertriglyceridemia: elevated blood triglyceride levels over 1.70 mmol/L in the presence of normal total cholesterol levels, or total cholesterol less than 572 mmol/L; (3) increased blood total cholesterol and triglyceride levels, or total cholesterol above 572 mmol/L and triglycerides over 170 mmol/L, are indicative of mixed hyperlipidemia; and (4) lipid control: participants with triglycerides and total cholesterol under 170 and 572 mmol/L, respectively, after lipid-lowering medication or exercise. Exclusion criteria include the following: (1) no more than one dose of the COVID-19 vaccine (BBIBP-CorV or Corona Vac), (2) age <18 years, (3) a history of SARS-CoV-2 infection, (4) the presence of immunosuppression or immunosuppression within 6 months, and (5) the presence of persistent pregnancy.

This study was approved by the Ethics Committee of the Second Affiliated Hospital of Chongqing Medical University and conformed with the ethical guidelines of the Declaration of Helsinki. Written informed consent was obtained from all participants prior to their inclusion in the study. This study has been registered at ClinicalTrials.gov (NCT05043246).

### 2.2 AEs

A questionnaire was used to gauge AEs 7 days following the vaccine. The Chinese Medical and Drug Administration’s scale was then used to classify all AEs (2019 version).

### 2.3 Abs’ detection

Using the fully automated chemiluminescence assay MAGLUMI 2000, we examined all plasma samples for S-RBD IgG antibody (anti-S-RBD IgG) and SARS-CoV-2 neutralizing antibodies (NAbs) (Snibe, Shenzhen, China). The kit’s instructions state that CoV-2 NAbs and anti-RBD IgG threshold values of >0.15 μg/mL and >1 AU/mL, respectively, are required for seropositivity, while a value of less than or equal to the threshold is required for seronegative status.

### 2.4 RBD^+^-specific B-cell assay

Fresh peripheral blood samples were collected and centrifuged through a Ficoll density gradient (Histopaque; Sigma-Aldrich Corporation, St Louis, MO, USA). Brilliant Violet 421™ Streptavidin-conjugated Abs (BioLegend, San Diego, CA, USA) and biotinylated Abs against the RBD of the SARS-CoV-2 spike protein (Sino Biological, Beijing, China) at a molar ratio of 1:4 were used for identification of the MBCs. For flow cytometry (Beckman Coulter, Inc., Brea, CA, USA), the cells were washed with phosphate-buffered saline, suspended staining buffer containing 2% fetal bovine serum, and probed with Abs against IgG, IgM, cluster of differentiation 3 (CD3), CD19, CD21, and CD27 (all, Biolegend). The data were examined using FlowJo software (version 10.0.7; FlowJo, LLC, Ashland, OR, USA). The cell percentages were calculated for just MBCs, include RBD-specific B cell (CD3− CD19+ RBD+), RBD-specific MBCs (CD3− CD19+ RBD^+^ CD27+), RBD^+^ atypical MBCs (CD3− CD19+ RBD^+^ CD21− CD27−), RBD^+^-activated MBCs (CD3− CD19+ RBD^+^ CD21− CD27+), RBD^+^-resting MBC (CD3− CD19+ RBD^+^ CD21+ CD27+), and RBD^+^ intermediate MBC (CD3− CD19+ RBD^+^ CD21 + CD27−).

### 2.5 Statistical analysis

Chi-square and Fisher’s exact tests were applied to categorical variables, while Mann–Whitney *U* test was applied to continuous variables with normally distributed or non-normal distribution, respectively. The Kruskal–Wallis test was used to compare three or more groups, and Bonferroni was employed to correct the outcomes of multiple comparisons. Using univariate and multivariate ordinal logistic regression analyses, the variables that significantly affected Abs were found. A statistical study was performed utilizing IBM SPSS (version 26.0). GraphPad Prism (version 9.2.0) was used for the plotting. The threshold for statistical significance was a *p* value of 0.05.

## 3 Results

As shown in Table 1, for HLD patients and HCs, no significant differences in median age, percentage of males, mean body mass index (BMI), vaccine type, median number of days after the second vaccination, results of routine blood tests (red blood cell, white blood cells, hemoglobin, and platelets), but mean BMI and lymphocyte were higher in HLD patients than in HCs.

**Table 1:** Characteristics of participants after two-dose vaccination

	HLD patients (*n* = 105)	HCs (*n* = 74)	*P*
Age (years)	59 (19–89)	61 (19–87)	0.814
**Gender**			
Male, *n* (%)	50.5% (53/105)	55.4% (41/74)	0.515
Female, *n* (%)	49.5% (52/105)	44.6% (33/74)	
BMI^#^ (kg/m^2^)	24.72 (19.28–48.83)	23.20 (13.60–33.98)	0.027
**Vaccine type**			
Corona	68.6% (72/105)	66.2% (49/74)	0.740
BBIBP-CorV	31.4% (33/105)	33.8% (25/74)	
Days between second dose and sample collection/antibody measurement, median (range)	50 (16–159)	41 (20–168)	0.239
Red blood cell^#^ (10^12^/L)	4.47 (2.37–5.66)	4.46 (2.46–6.50)	0.789
Hemoglobin^#^ (g/L)	137 (71–171)	137 (73–179)	0.924
White blood cell^#^ (10^9^/L)	6.37 (2.67–15.44)	6.09 (3.11–11.47)	0.101
Lymphocyte^#^ (10^9^/L)	1.69 (0.45–5.91)	1.55 (0.21–2.70)	0.029
Platelet^#^ (10^9^/L)	199 (101–366)	205 (94–420)	0.846

Lists the participant characteristics after the full vaccination cycle. Fisher’s exact test and the Chi-Square statistic test were applied to categorical variables, while the Mann-Whitney *U* test was applied to continuous variables. At *p* < 0.05, statistics were deemed significant.

^#^Shown as the median (range).

As can be shown in Table 2, there were no appreciable differences in the AEs that occurred 7 days following COVID-19 vaccination between HLD patients and HCs.

**Table 2:** AEs of COVID-19 vaccination in participants

Variable	HLD patients (*n* = 105)	HCs (*n* = 74)	*P*
Overall AEs within 7 days	13.3% (14/105)	9.5% (7/74)	0.428
Local AEs	7.6% (8/105)	4.1% (3/74)	0.508
Systemic AEs	5.7% (6/105)	5.4% (4/74)	1.000
Grade 3 and 4 AEs	/	/	/

Data are expressed as *n* (%) and the chi-square test was used to compare the statistical differences between the two groups.

### 3.1 Humoral immune response to inactivated SARS-CoV-2 vaccines in HLD patients

**Figure 1:**
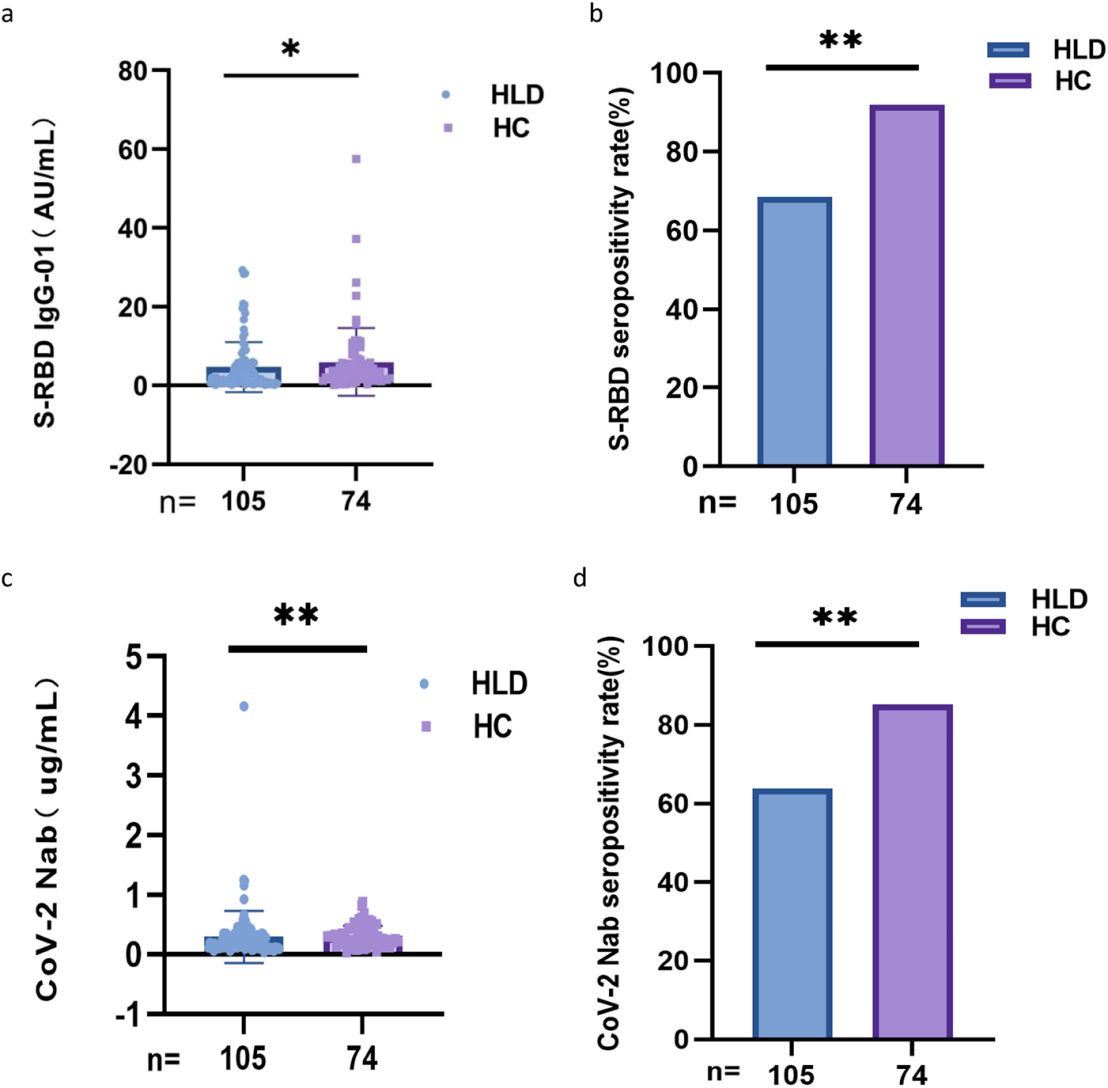
Antibodies response to inactivated SARS-CoV-2 vaccines in HLD patients. (a and b) The seropositivity rates and titers of anti-RBD IgG in HLD patients. (c and d) The seropositivity rates and titers of CoV-2 NAbs in HLD patients. The Chi-square test and Mann–Whitney *U* test were used for comparison.

As shown in Figure 1, seropositivity rates (68.9% vs 91.9%, *p* < 0.001, 64.2% vs 85.1%, *p* = 0.002) and titers (median [interquartile range (IQR)]: 2.24 [0.67–5.30] vs 3.35 [1.83–6.05], *p* = 0.026, 0.19 [0.12–0.32] vs 0.24 [0.19–0.43], *p* = 0.002) for the both Abs (anti-RBD IgG and CoV-2 NAbs) were lower in HLD patients than in HCs.

**Figure 2:**
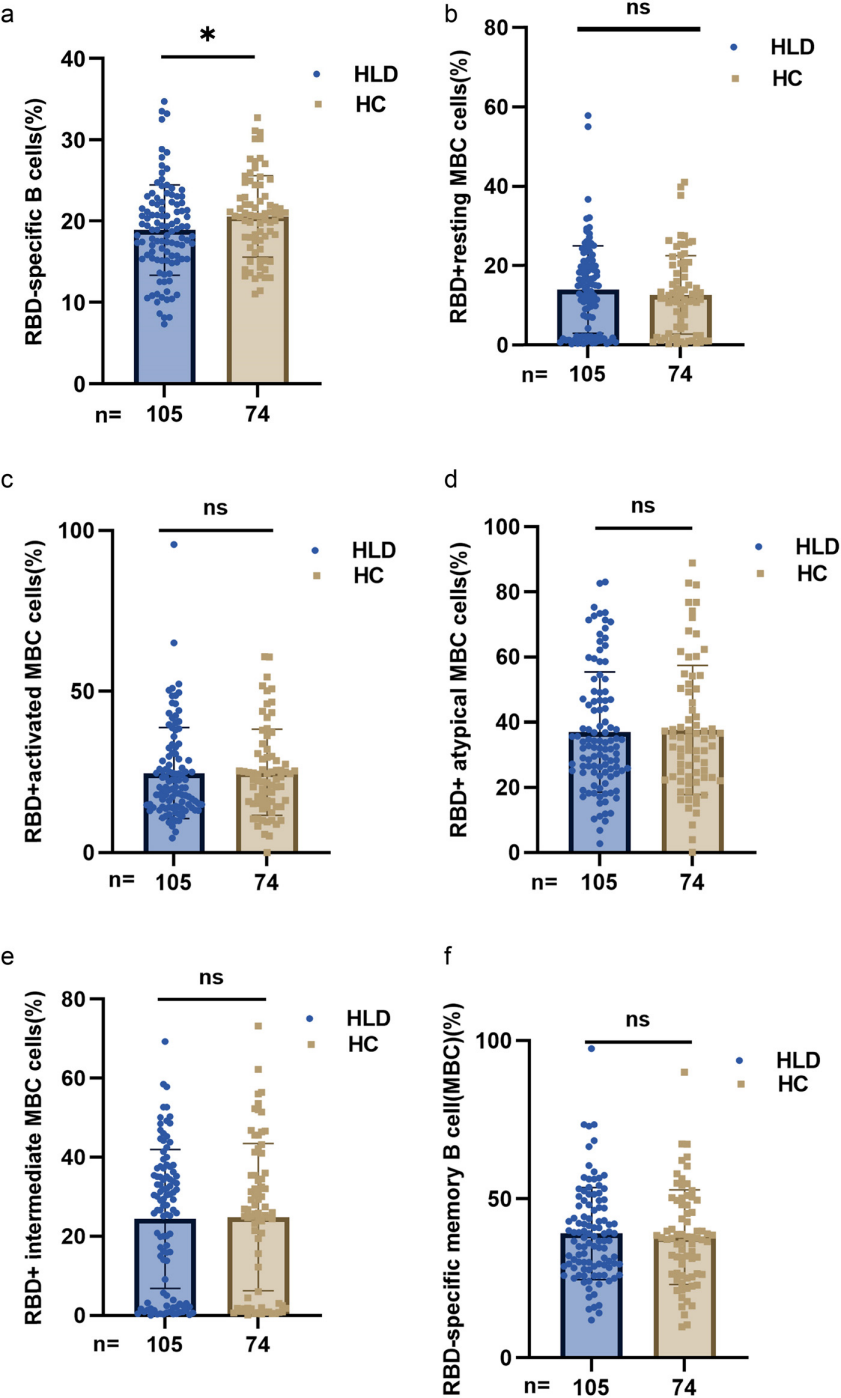
MBC response to inactivated SARS-CoV-2 vaccines in HLD patients. (a–f) The frequencies of RBD-specific B cells, RBD^+^-resting MBCs, RBD^+^-activated MBCs, RBD^+^ atypical MBCs, RBD^+^ intermediate MBCs, and RBD^+^-specific MBCs in HLD patients, and the Mann–Whitney *U* test was used for comparison.

In contrast to HCs, HLD patients had reduced frequencies of RBD-specific B cells (mean [95% confidence interval (CI)]: 18.88 [17.79–19.94] vs 20.63 [17.31–23.35], *p* = 0.040), as shown in Figure 2. The frequencies of RBD^+^-resting MBCs, RBD^+^-activated MBCs, RBD^+^ atypical MBCs, RBD^+^ intermediate MBCs, and RBD^+^-specific MBCs were similar in HLD patients and HCs.

### 3.2 Humoral immune response to inactivated SARS-CoV-2 vaccines in HLD subgroups

**Figure 3:**
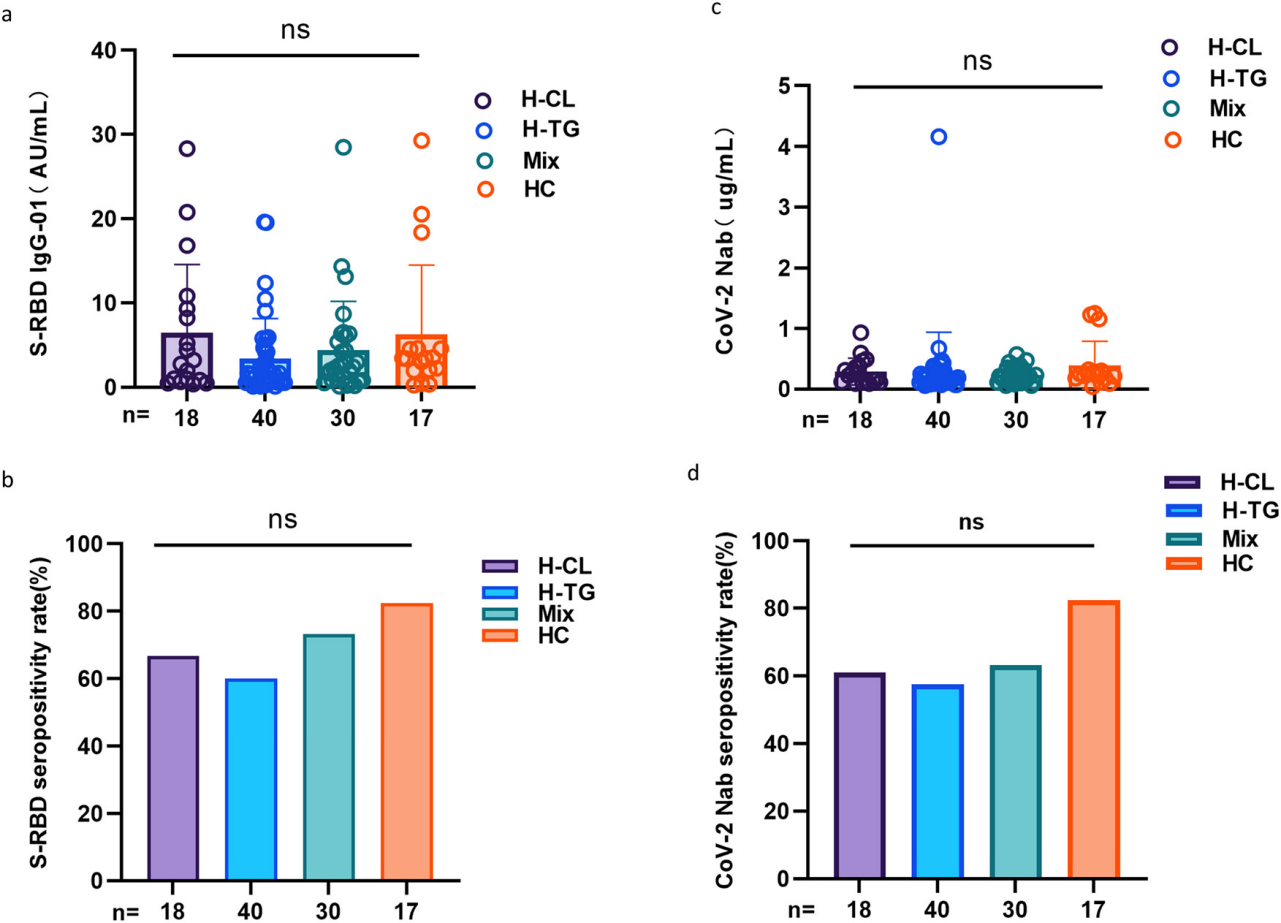
Antibodies response to inactivated SARS-CoV-2 vaccines in HLD subgroups. (a and b) The seropositivity rates and titers of anti-RBD IgG in the hyperlipidemic group (H-CL), high triglyceride group (H-TG), mixed group (MiX), and lipid control group (HC). (c and d) The seropositivity rates and titers of CoV-2 NAbs in the hyperlipidemic group (H-CL), high triglyceride group (H-TG), mixed group (MiX), and lipid control group (HC). The Chi-square test and Kruskal–Wallis test were used for comparison.

Figure 3 illustrates the seropositivity rates and titers of the both Abs among in H-CL, H-TG, MiX, and HC. Seropositivity rates (66.7% vs 60.0% vs 73.3% vs 82.4%, *p* = 0.361, 61.1% vs 57.5% vs 63.3% vs 82.4%, *p* = 0.350) and titers (2.99 [0.80–9.68] vs 1.66 [0.57–4.56] vs 2.45 [0.85–6.13] vs 3.45 [2.01–4.60], *p* = 0.244, 0.24 [0.18–0.40] vs 0.17 [0.11–0.30] vs 0.20 [0.12–0.35] vs 0.25 [0.18–0.31], *p* = 0.360) of the both Abs were not significantly different among these groups.

**Figure 4:**
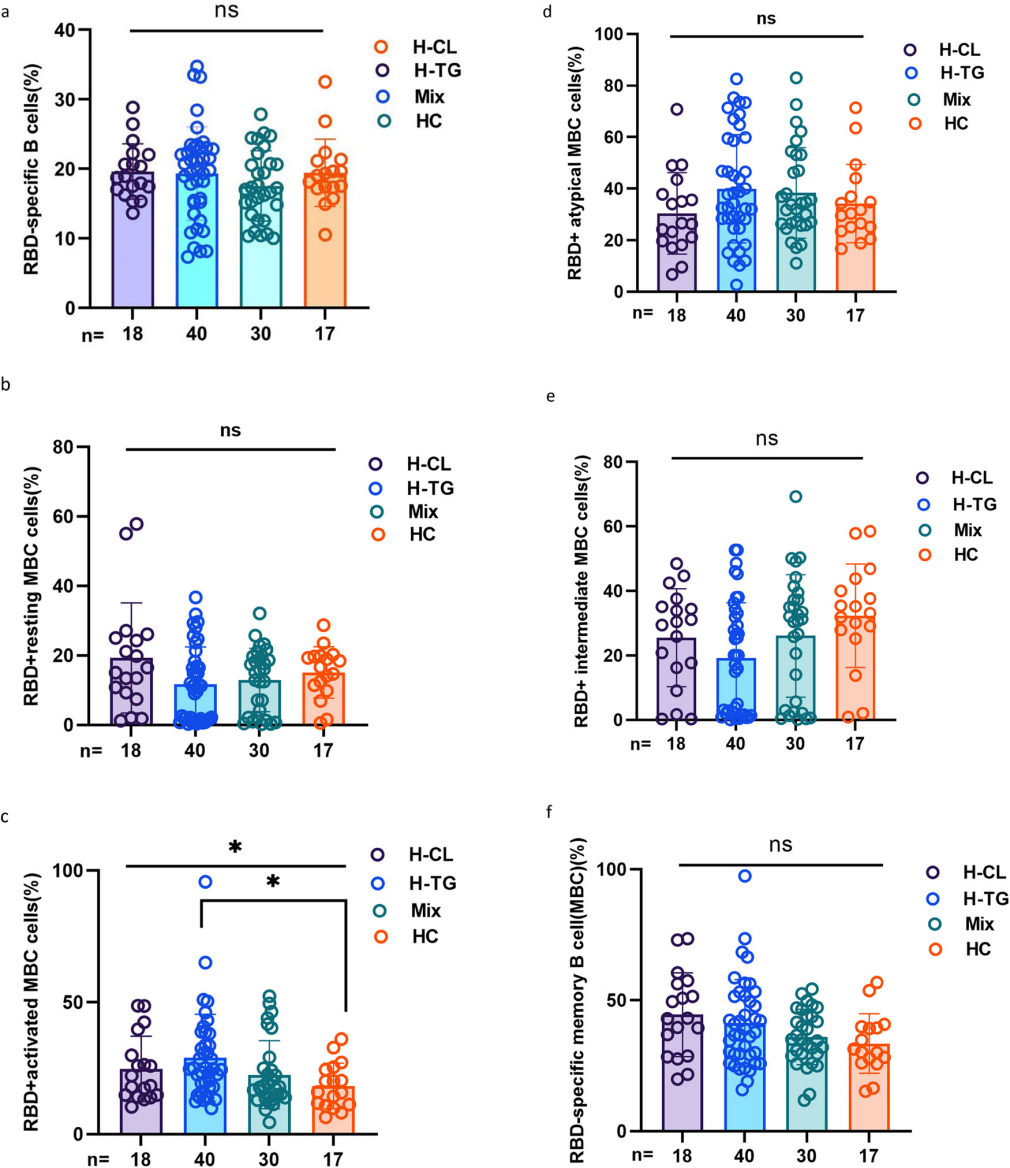
MBCs’ response to inactivated SARS-CoV-2 vaccines in HLD subgroups. (a–f) The frequencies of RBD-specific B cells, RBD^+^-resting MBCs, RBD^+^-activated MBCs, RBD^+^ atypical MBCs, RBD^+^ intermediate MBCs, and RBD^+^-specific MBCs in the high cholesterol group (H-CL), high triglyceride group (H-TG), mixed group (MiX), and lipid control (HC). The Kruskal–Wallis test was used for comparison, and the Bonferroni test was used to rectify it.

As shown in Figure 4, the frequencies of RBD-specific B cells, RBD^+^-resting MBCs, RBD^+^ atypical MBCs, RBD^+^ intermediate MBCs, and RBD^+^-specific MBCs were not significantly different among the H-CL, H-TG, MiX, and HC. Only RBD^+^-activated MBC (20.30 [14.73–32.38] vs 24.75 [18.00–37.03] vs 17.90 [13.83–26.03] vs 16.00 [11.05–24.96], *p* = 0.037) differed among the four groups.

### 3.3 Humoral immune response to inactivated SARS-CoV-2 vaccines in HLD patients aged ≥65 or <65 years

**Figure 5:**
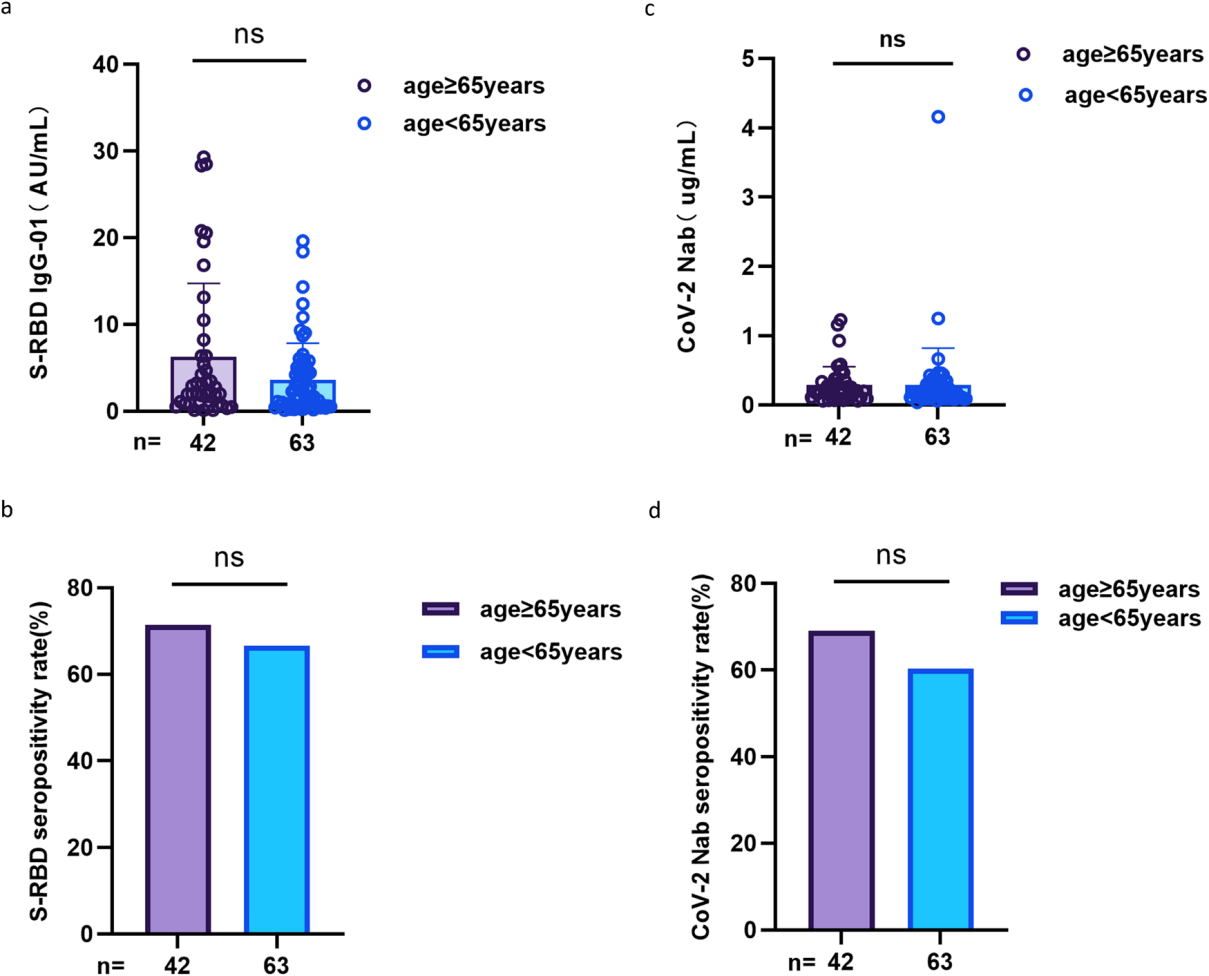
Antibodies’ response to inactivated SARS-CoV-2 vaccines in HLD patients aged ≥65 or <65 years. (a and b) The seropositivity rates and titers of anti-RBD IgG for HLD patients aged ≥65 or <65 years. (c and d) The seropositivity rates and titers of CoV-2 NAbs for those aged ≥65 or <65 years. The Chi-square test and Mann–Whitney *U* test were used for comparison.

According to Figure 5, there were no significant differences in seropositivity rates (71.4% vs 66.7%, *p* = 0.607, 69% vs 60.3%, *p* = 0.362) and titers (median [IQR]: 2.46 [0.84–6.82] vs 2.24 [0.60–4.63], *p* = 0.282, 0.21 [0.11–0.34] vs 0.18 [0.12–0.32], *p* = 0.598) for both Abs in HLD patients aged ≥65 or <65 years.

**Figure 6:**
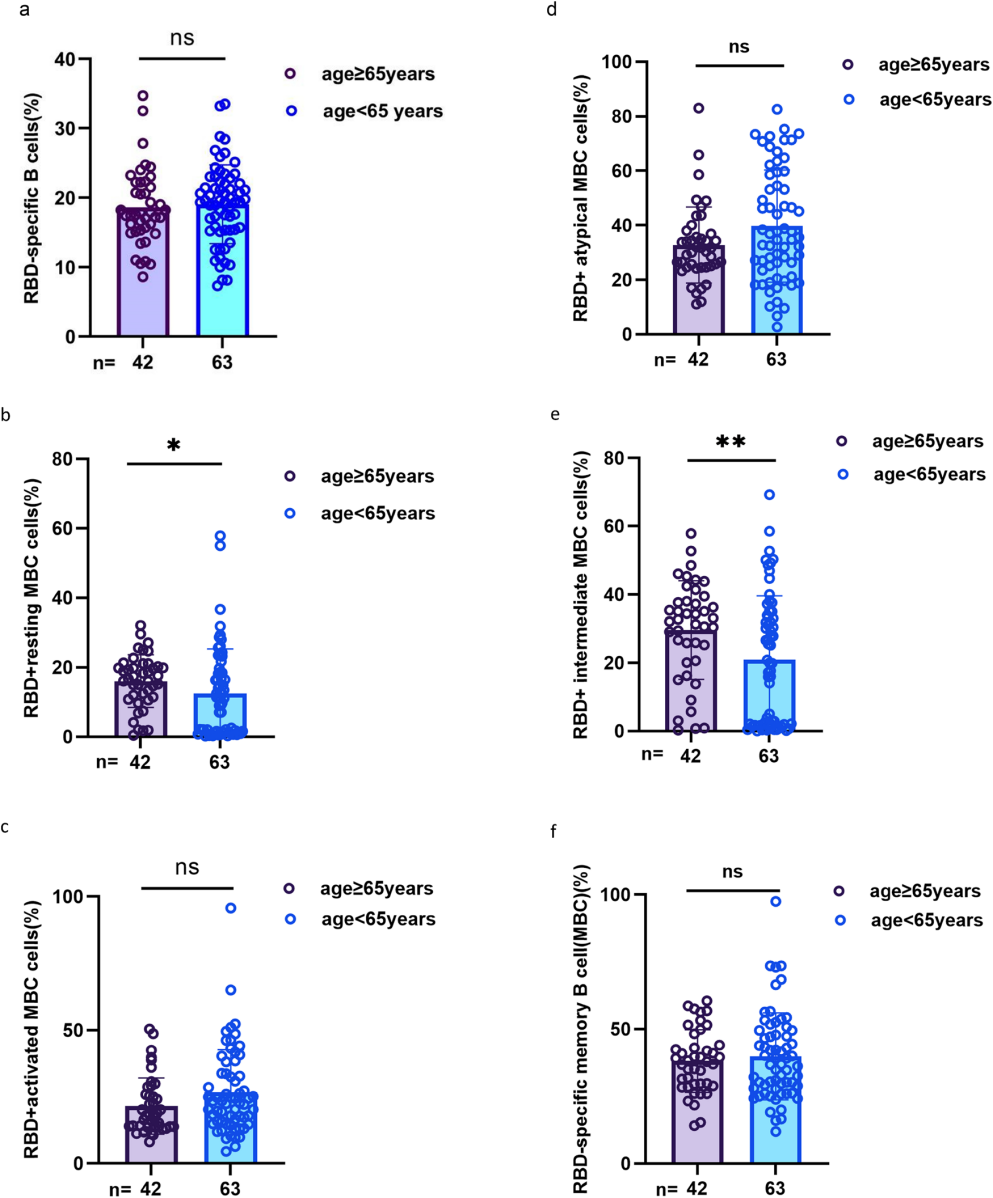
MBCs’ response to inactivated SARS-CoV-2 vaccines in HLD patients aged ≥65 or <65 years. (a–f) The frequencies of RBD-specific B cells, RBD^+^-resting MBCs, RBD^+^-activated MBCs, RBD^+^ atypical MBCs, RBD^+^ intermediate MBCs, and RBD^+^-specific MBCs in HLD patients aged ≥65 or <65 years, and the Mann–Whitney *U* test was used for comparison.

As shown in Figure 6, the frequencies of RBD^+^-resting MBCs and RBD^+^ intermediate MBCs were lower in HLD patients aged ≥65 years than in <60 years (median [IQR]: 17.00 [11.33–20.43] vs 11.27 [1.21–18.60], *p* = 0.011, 32.40 [20.48–38.38] vs 20.00 [1.72–33.90], *p* = 0.009). In contrast, there were no significant differences in RBD-specific B cells, RBD^+^ atypical MBCs, RBD^+^-activated MBCs, and RBD^+^-specific MBCs.

As shown in Tables 3 and 4, we found that days between the second dose and sample collection/antibody measurement were associated with lower anti-RBD IgG Ab levels through univariate and multivariate linear regression analyses.

**Table 3:** Univariate and multifactorial analyses of anti-RBD IgG in HLD patients

	Univariate OR (95% CI)	*P*	Multivariate OR (95% CI)	*P*
Gender (female)	0.659 (0.283, 1.507)	0.326	1.198 (0.224, 0.698)	0.833
Age (years)	1.015 (0.987, 1.044)	0.308	1.022 (0.954, 1.072)	0.704
BMI (kg/m^2^)	0.993 (0.889, 1.120)	0.900	0.967 (0.824, 1.146)	0.663
Vaccine tape (Corona Vac)	1.690 (0.703, 4.029)	0.236	1.536 (0.346, 6.881)	0.567
Days between second dose and sample collection/antibody measurement	0.969 (0.954, 0.983)	<0.001	0.975 (0.950, 0.996)	0.032
Red blood cell (10^12^/L)	0.879 (0.393, 1.913)	0.746		
White blood cell (10^9^/L)	0.937 (0.753, 1.168)	0.551		
Hemoglobin (g/L)	1.006 (0.980, 1.033)	0.638		
Lymphocyte (10^9^/L)	1.129 (0.623, 2.244)	0.704		
Platelet (10^9^/L)	1.000 (0.992, 1.008)	0.985		
Cholesterol (mmol/L)	1.115 (0.745, 1.705)	0.601	1.161 (0.684, 2.008)	0.572
Triglyceride (mmol/L)	1.132 (0.818, 1.659)	0.481	1.022 (0.590, 1.967)	0.940
RBD-specific B cells (%)	1.008 (0.936, 1.088)	0.832	1.032 (0.896, 1.192)	0.654
RBD^+^-resting MBC (%)	1.033 (0.993, 1.080)	0.123	0.517 (1.187, 1.723)	0.930
RBD^+^-activated MBC (%)	0.983 (0.954, 1.012)	0.241	0.475 (0.106, 1.587)	0.921
RBD^+^ atypical MBC (%)	0.987 (0.965, 1.009)	0.246	0.103 (0.028, 2.879)	0.760
RBD^+^ intermediate MBC (%)	1.015 (0.991, 1.040)	0.230	0.101 (0.027, 2.853)	0.759
RBD-specific MBC (%)	1.000 (0.972, 1.030)	0.999	0.214 (0.088, 3.729)	0.300

CI, confidence interval, OR, odds ratio, RBD, receptor-binding domain, MBC, memory B cell.

## 4 Discussion

In this prospective trial, we assessed the inactivated SARS-CoV-2 vaccine’s safety, Ab responses, RBD-specific B cells, and MBCs in HLD patients and HCs. Our findings demonstrate that the inactivated vaccination is safe and well tolerated in HLD patients but reduced the titers of Abs and the frequencies of RBD-specific B cell.

**Table 4:** Univariate and multifactorial analysis of CoV-2 NAbs in HLD patients

	Univariate OR (95% CI)	*P*	Multivariate OR (95% CI)	*P*
Gender (female)	0.626 (0.278, 1.392)	0.254	0.720 (0.156, 3.330)	0.669
Age (years)	1.015 (0.987, 1.043)	0.296	0.994 (0.942, 1.046)	0.807
BMI (kg/m^2^)	1.006 (0.903, 1.133)	0.913	1.029 (0.887, 1.205)	0.694
Vaccine tape (Corona Vac)	1.775 (0.759, 4.154)	0.183	1.515 (0.395, 5.827)	0.540
Days between second dose and sample collection/antibody measurement	0.977 (0.963, 0.989)	<0.001	0.984 (0.962, 1.004)	0.136
Red blood cell (10^12^/L)	1.213 (0.570, 2.608)	0.614		
White blood cell (10^9^/L)	0.940 (0.759, 1.164)	0.566		
Hemoglobin (g/L)	1.015 (0.990, 1.043)	0.242		
Lymphocyte (10^9^/L)	0.895 (0.498, 1.624)	0.702		
Platelet (10^9^/L)	1.001 (0.994, 1.009)	0.768		
Cholesterol (mmol/L)	1.138 (0.780, 1.696)	0.507	1.141 (0.702, 1.906)	0.594
Triglyceride (mmol/L)	1.012 (0.743, 1.409)	0.939	1.276 (0.742, 2.587)	0.432
RBD-specific B cells (%)	1.007 (0.937, 1.083)	0.857	1.046 (0.925, 1.193)	0.475
RBD^+^-resting MBC (%)	1.042 (1.002, 1.089)	0.051	0.485 (0.117, 1.550)	0.910
RBD^+^-activated MBC (%)	0.978 (0.948, 1.006)	0.125	0.452 (0.106, 1.455)	0.902
RBD^+^ atypical MBC (%)	0.977 (0.955, 0.999)	0.043	0.536 (0.156, 1.652)	0.922
RBD^+^ intermediate MBC (%)	1.027 (1.003, 1.052)	0.031	0.540 (0.157, 1.684)	0.923
RBD-specific MBC (%)	0.999 (0.972, 1.028)	0.971	1.173 (0.074, 1.919)	0.907

CI, confidence interval, OR, odds ratio, RBD, receptor-binding domain, MBC, memory B cell.

COVID-19 patients with dyslipidemia may worsen mortality and severity [1,2,13], hence prompt administration of the inactivated vaccine may be advantageous in this population of patients. Limited registration trials have been conducted, nonetheless, on the humoral immune response and safety of the inactivated SARS-CoV-2 vaccination in individuals with HCs. The overall incidence of AEs within 7 days of vaccination with the COVID-19 vaccine in HLD patients was reported to be 13.3%, comparable to HCs (9.5%), when we initially evaluated the safety of inactivated vaccine in HLD patients, but lower than in phase 3 trials of the Turkish [14] Corona vaccine (18.9%) and phase 1/2 studies of the Chinese [15] BBIBP-CorV (23–29%). The HLD patients and HCs experienced similar local AEs (7.6% vs 4.1%) and overall AEs (5.7% vs 5.4%), and no significant AEs (grade 3/4 AEs) occurred in any of the participants.

Our results showed that the titers of both Abs were lower in HLD patients than in HCs 16–168 days after vaccination; previous study has demonstrated that Ab levels are lower in obese patients than in healthy groups after vaccination [7,8,16], and this study’s HLD patients’ BMI was also higher than that of HCs. Obesity is strongly associated with HLD. Patients with HLD also had reduced seroconversion rates for both Abs, at 68.6 and 63.5%, respectively. However, by mixed factors analysis, no correlation was found between Ab titers and lipid or cholesterol concentrations. The reason may be that the overall vaccination time of this patient cohort is longer or immunogenicity itself is lower.

Our findings revealed that the frequencies of RBD-specific B cells were lower in HLD patients than in HCs, and prior research revealed that the frequencies of RBD-specific B cells were significantly lower in some patients with chronic disease 1 month after receiving the inactivated COVID-19 vaccine [17]. These findings suggested that humoral immunity in HLD patients may be compromised after receiving the SARS-CoV-2 vaccine.

In our result that we found the frequencies of RBD^+^-activated MBCs were lower in lipid control group than in high triglyceride group, but not for other MBCs, may be due to the MBCs were resting or intermediate status in lipid control group.

Similar to the findings of some earlier studies [18,19,20], we used mixed factor analysis to again identify days between the second dose and sample collection/antibody measurement as a risk factor for anti-RBD IgG Abs’ responses. This suggests the need for monitoring the Ab levels and booster dose in time. Contrary to the findings of earlier research [21,22,23], etc., age and gender were not related to Ab levels in our investigation. This analysis may have been necessary because of the study’s limited sample size, which has to be further confirmed by further trials with a larger sample size.

There are some flaws in the current study. First of all, this study was conducted in a single center with a modestly sized sample. Second, the T cells were not analyzed in the study. Third, there were no longitudinal analyses; only cross-sectional comparisons were made. But this research has certain advantages as well: For the first time, the safety and immunogenicity of COVID-19 vaccine for patients with hyperlipemia have been clarified and helped doctors respond to patient concerns. As for safety, Ab response, RBD-specific B cells, and MBCs, this study gives an adequate evaluation of all of these factors. Third, days between the second dose and sample collection/antibody measurement were found to be a risk factor for anti-RBD IgG Ab levels, meaning that Ab levels dropped with time.

In conclusion, patients with HLD tolerated the SARS-CoV-2-inactivated vaccine well and without any severe AEs, but the humoral immune may be limited.

## Figures and Tables

**Figure A1:**
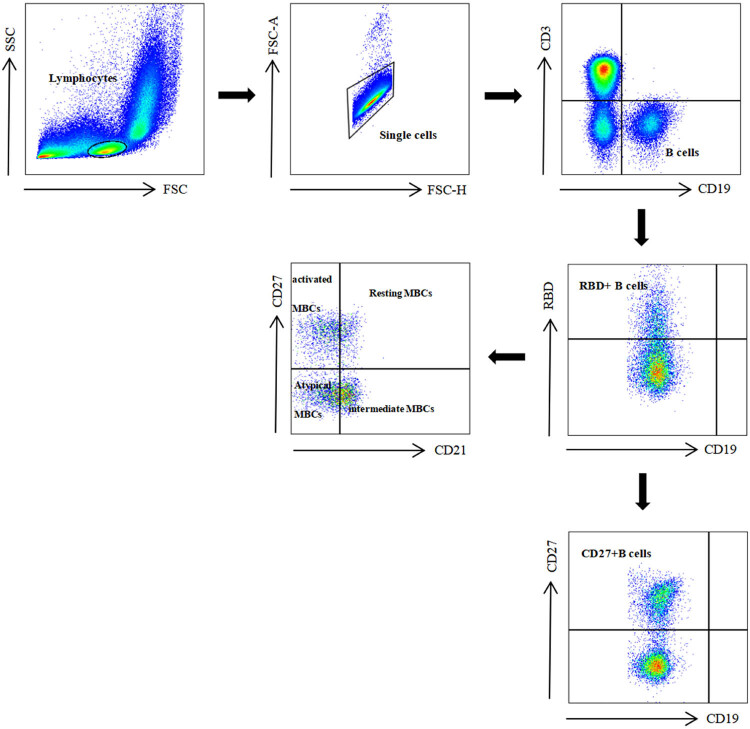
Full gating strategy of flow cytometry for target cell population.
